# Accurate and lightweight MRI super-resolution via multi-scale bidirectional fusion attention network

**DOI:** 10.1371/journal.pone.0277862

**Published:** 2022-12-15

**Authors:** Ling Xu, Guanyao Li, Qiaochuan Chen

**Affiliations:** 1 Tongji University, College of Electronics and Information Engineering, Jiading District, Shanghai, China; 2 Shanghai University, College of Computer Engineering and Science, Baoshan District, Shanghai, China; University of Engineering & Technology, Taxila, PAKISTAN

## Abstract

High-resolution magnetic resonance (MR) imaging has attracted much attention due to its contribution to clinical diagnoses and treatment. However, because of the interference of noise and the limitation of imaging equipment, it is expensive to generate a satisfactory image. Super-resolution (SR) is a technique that enhances an imaging system’s resolution, which is effective and cost-efficient for MR imaging. In recent years, deep learning-based SR methods have made remarkable progress on natural images but not on medical images. Most existing medical images SR algorithms focus on the spatial information of a single image but ignore the temporal correlation between medical images sequence. We proposed two novel architectures for single medical image and sequential medical images, respectively. The multi-scale back-projection network (MSBPN) is constructed of several different scale back-projection units which consist of iterative up- and down-sampling layers. The multi-scale machine extracts different scale spatial information and strengthens the information fusion for a single image. Based on MSBPN, we proposed an accurate and lightweight Multi-Scale Bidirectional Fusion Attention Network(MSBFAN) that combines temporal information iteratively. That supplementary temporal information is extracted from the adjacent image sequence of the target image. The MSBFAN can effectively learn both the spatio-temporal dependencies and the iterative refinement process with only a lightweight number of parameters. Experimental results demonstrate that our MSBPN and MSBFAN are outperforming current SR methods in terms of reconstruction accuracy and parameter quantity of the model.

## Introduction

Magnetic resonance imaging (MRI) is a non-invasive medical imaging technique that offers outstanding spatio-temporal resolution and clear soft-tissue been contrast. Since its invention in 1972, MRI has proven to be a versatile imaging technique and is widely used in hospitals and clinics. Compared with other imaging techniques such as computed tomography (CT) and positron emission tomography (PET), MRI does not involve X-rays or the use of ionizing radiation. However, clinically, to acquire high-quality MR images, patients usually are needed to remain stable in a narrow tube for a long time, which aggravates the patient’s discomfort and unavoidably introduces motion artefacts that compromise image quality. Long acquisition times and the sustained increase in demand for MRI within health systems has led to concerns about cost-effectiveness.

To accelerate the acquisition speed and ensure the quality of the MR image, a large number of published studies consider adopting super-resolution (SR) algorithms without any hardware update, which have been widely studied and applied in the natural image domain. The image SR algorithm base on interpolation estimates the value of the current pixel through the adjacent pixels [1, 2]. By combining prior information, the image SR algorithm based on reconstruction generates the high-resolution image [3]. The proposition of compressed sensing (CS) proved that the sparsity of a signal could be exploited to recover it from far fewer samples than required by the Nyquist-Shannon sampling theorem. Past literature based on CS has achieved preferable performance in MR imaging [4, 5]. Lingala et al. [6] show that exploiting spatio-temporal redundancy from sequence MR images can immensely improve image reconstruction quality. However, one of the most significant challenges of those traditional approaches is that the reconstructed image introduces smoothness and aliasing artefacts that work so intensely to the disadvantage of image quality. Furthermore, the regularization functions and their hyper-parameters are sensitive and must be selected carefully, which brings great difficulty to practical application.

In recent years, multiple advanced SR models [7–10] have been proposed with the significant development of deep learning and attracted increasing attention due to their superior performance on natural images. Unlike traditional algorithms, deep learning methods directly learn an end-to-end mapping between the low/high-resolution image-pairs without specifying the point information and regularization in the training process. Dong et al. [11] is the pointing work that introduces convolutional neural networks (CNNs) [12] to the SR field, which confirms the advantage of CNN in image feature extraction. The following works mainly focuse on developing model depth and width to construct more complex structures that have better extract and merge feature maps. However, those methods are mainly aimed at natural image SR rather than medical image. Meanwhile, training and applying the deeper and wider models is difficult due to the great numbers of parameters and much computing resources.

Benefiting from the convenience of medical image dataset acquisition, researchers employed various kinds of neural networks to enhance the quality of MR images directly [13–19]. Those deep learning-based methods assimilate the characteristics of MR images and have better performance than natural image models. Although existing medical image SR methods have achieved significant improvements, they still suffer from several limitations. Firstly, almost all existing methods only extract information at a single scale and neglect the information of the other scales, which often take a lot of parameters due to the large kernel size and damage the accuracy of the model. Secondly, most existing deep learning research for MR images is based on a single image or different image sequences, which exploit inherent image redundancy to recover lost high-frequency details but ignore the temporal correlations of the medical image sequence. There is no complete exploitation of spatio-temporal dependencies. Finally, for better SR performance, the SR models are getting more and more complexity. However, non-attention methods [20–22] treat all image features equally, which prevents training deeper models and is detrimental to image reconstruction. Meanwhile, for sequence medical images, the abundant spatial information features of the target slice and the supplementary temporal information features from a set of adjacent slices have different effects on target slice reconstruction.

In this paper, we propose two novel networks to resolve the remaining issues mentioned above. For a single medical image, we present a multi-scale back-projection network (MSBPN) to extract the information from different scales, which is beneficial to reduce the number of parameters and further improve the SR performance. For sequence medical images, we integrate the benefits of the MSBPN and propose an accurate and lightweight multi-scale bidirectional fusion attention network (MSBFAN) to explore the spatio-temporal dependencies iteratively. Specifically, we employ MSBPN to explore the abundant spatial information of the target slice, and adopt ResNet [23]to extract the supplementary temporal information from a set of adjacent slices, then fusion attention were employed to filter and combine the spatial and temporal information to improve the quality of the target slice further. Our contributions include the following key innovations:

**Multi-Scale Back-Projection Network for single target MR image:** We propose MSBPN for extracting details of different scales through multiple up- and down-sampling layers. We combine back-projection and multi-scale to expose residual features of multiple scales, and thus better performance and computational efficiency are achieved.

**Iteratively integrating spatial and temporal information:** For sequence MR images, spatial and temporal information is extracted from different sources. Spatial exploration block outputs various feature maps of the target slice and temporal exploration block extracts multiple sets of feature maps from adjacent slices. Those different sources are fused into the HR slice iteratively. To our best knowledge, and MR image SR, this is the first work to adequately investigate the temporal information supplement of the target slice from the adjacent slice.

**Fig 1:**
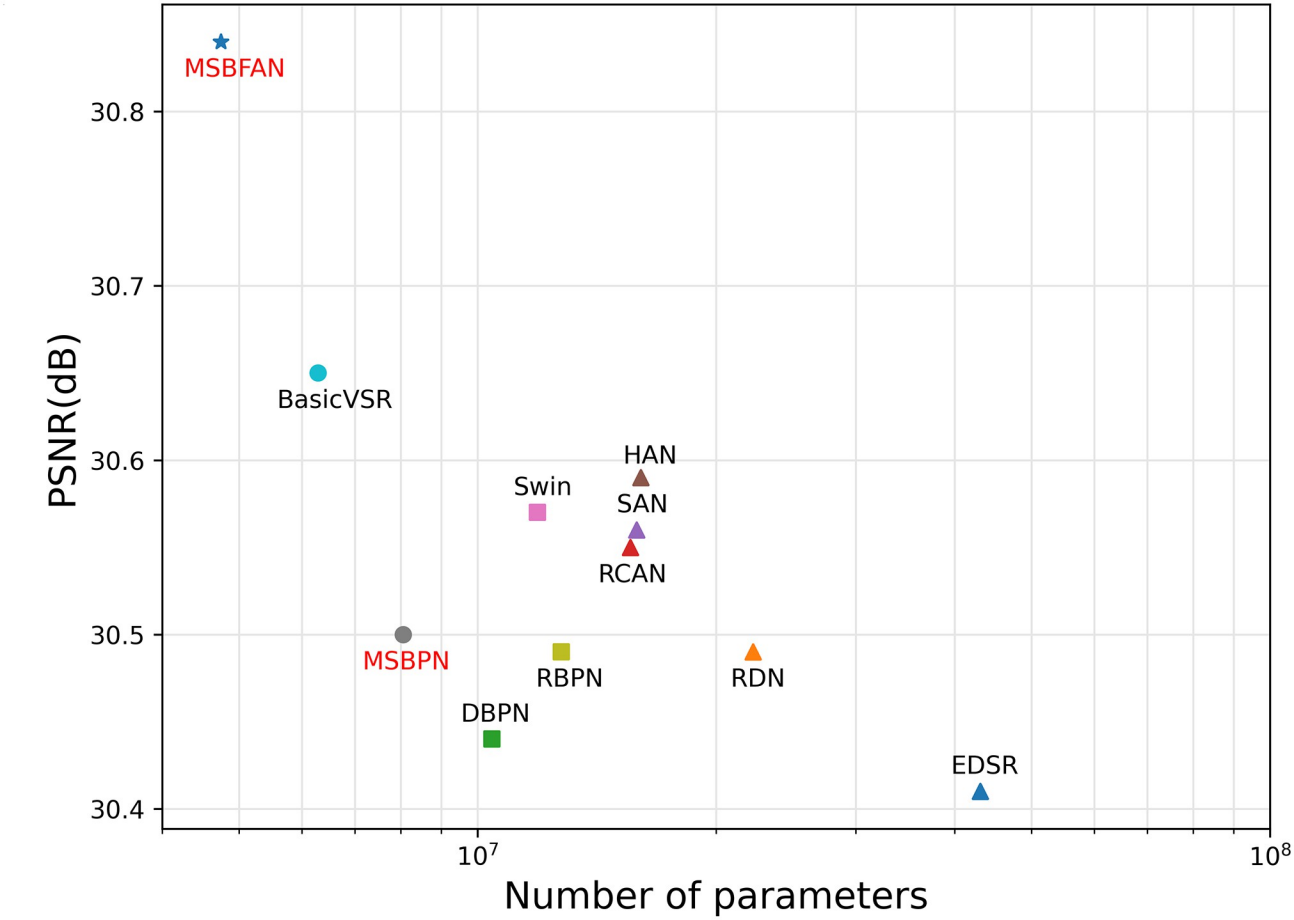
The performance comparison between several SR model on IXI dataset for SR x4. The test results vs. the number of model parameters. The symbols ⋆, ∘, □ and △ represent models with less than 5M, 10M, 15M and more than15M parameters respectively. Note that RBPN, BasicVSR and our MSBFAN treat 7 slices of a 3D volume as one training sample.

**Multi-Scale Bidirectional Fusion Attention Network for sequence MR image:** We propose MSBFAN, a bidirectional recurrent neural network based on MSBPN, which use only a modest number of parameters to achieve the state-of-the-art performance on SR task (Fig 1). Our MSBFAN effectively boost the performance via iteratively integrating spatial information and temporal information.

## Related work

The key problem of image super-resolution is how to perform upsampling [24]. Based on the employed upsampling operations and their locations, the architectures of existing models can be divided into following types. Pre-upsampling models [11] also utilize traditional upsampling algorithms to obtain middle higher-resolution images and then refine them using residual learning [25, 26] and recursive layers [27]. Pre-upsampling makes the model learning much easier, however, this approach often introduces extra noise and blurring, while increasing the cost of time and space. Post-upsampling [22, 28–32] performs the most computation in low-dimensional space to improve the computational efficiency and increase resolution automatically at the end of models. However, those models also require a large number of parameters due to failure to learn complicated mapping. Progressive upsampling [24, 33] is based on the cascade of upsampling modules to decompose a complex task into several simple tasks and progressively reconstruct multiple SR images, which dramatically reduces the learning difficulty. Iterative up- and down-sampling [20, 34, 35] apply back-projection [36] to compute the reconstruction error then fuse it back to tune the HR image intensity. This framework can better explore the deep relationships between LR-HR image pairs. Recently, some works [37–41] have adopted multi-scales to fully exploit the image feature, but there is no research on the fusion of iterative projection and multi-scale.

Benefit by the development of deep learning, more and more researchers have presented 2D and 3D CNNs models for medical images. As we know, 3D CNNs also outperform 2D CNNs in spatio-temporal feature extracting. However, 3D CNNs are more difficult to train due to the small number of high-quality training samples and many parameters. Schlemper et al. [16] used a deep cascade of CNNs to reconstruct dynamic sequences of 2D cardiac MR images. Qin et al. [15] combined traditional iterative algorithms with CNN then proposed a convolutional recurrent neural network. Zhao et al. [18] proposed a deep channel splitting network (CSN) which has two branches used for different information transmissions. Qing et al. [17] found that combining multi-contrast information contributes to reconstructing the results. Zhang et al. [42] proposed a queeze and excitation reasoning attention networks for accurate 2D MR. Although those 2D CNNs models have shown excellent ability to reconstruct the 2D MR images, they still lack the ability to extract the temporal information.To extract temporal features of the 3D MR volume more fully, 3D CNNs models extract features from 3D MR volume directly. One kind of 3D CNNs model [43] converts existing state-of-the-art deep 2D super-resolution models into 3D versions and improves some structures, which is the most convenient and fastest way to apply 3D CNNs to MR images. However, those kinds of models struggle to strike a balance between the number of parameters and performance. Recently, Li et al. [44] presented a ParalleNet using parallel connections and group convolution to treat features on different channels unequally. The number of network parameters and computational complexity can be reduced significantly while maintaining accuracy.

## Proposed method

### Multi-scale back-projection

**Fig 2:**
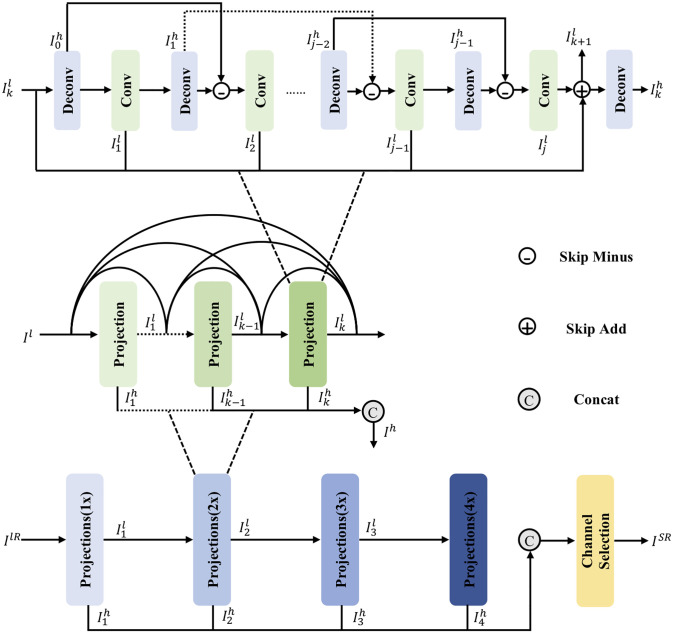
The diagram of the proposed MSBPN model. The overall structure consists of two main parts: cascaded projections of different scales and a channel selection layer. Each scale projection module is composed of *k* sub-projection modules that employ densely connected to encourage feature reuse. Each sub-projection module contains *j* alternating up- and down-layer group to generate the projection error and iteratively refine the LR.

In the case of 4x, as shown in Fig 2, we construct an end-to-end trainable architecture based on four scales (1x, 2x, 3x and 4x), then a channel selection mechanism will select and output the concatenated HR features. Where *k* = 1, 2, …, *n* and *j* = 2, 3, …, *m* are the index of the *k* − *th* projection unit in each scale and the *j* − *th* layer in the projection unit. Stacked up- and down-sampling layers output the synthesized LR feature for each scale and map it to HR features, such that the detail of different scales can be fused into the HR image. In such a design, the MSBPN integrates different scale detail, further improves the performance, and simplifies our model’s parameter.

### Overall network architecture

**Fig 3:**
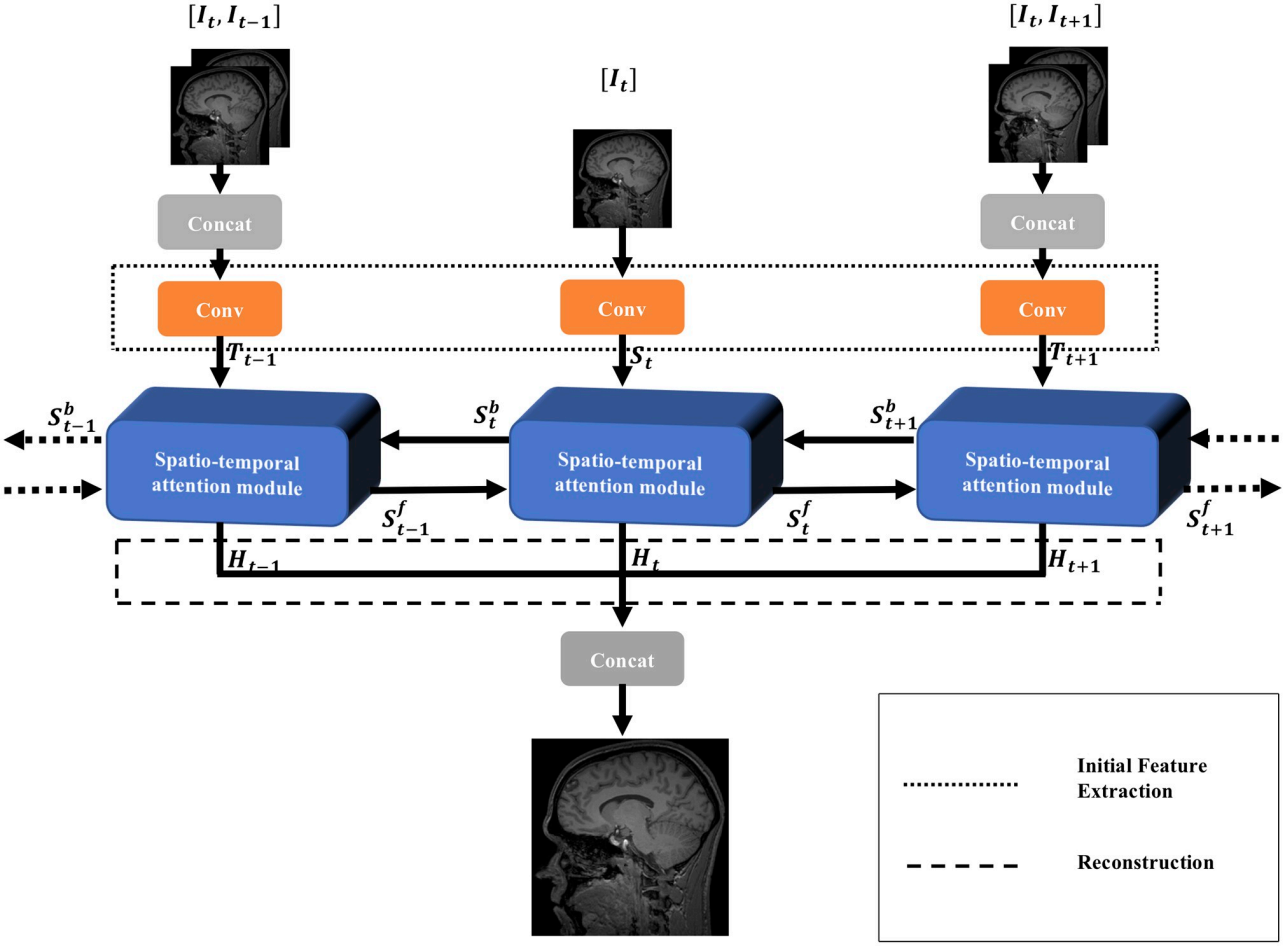
Overview of the proposed MSBFAN for sequence MR image SR. The overall structure consists of three parts: Initial feature extraction *F*_*E*_(.), spatio-temporal module *F*_*STAM*_(.) and reconstruction *F*_*R*_(.). The horizontal line is based on our MSBPN to explore the spatial information of target slice. The vertical line computes the residual features from a pair of target and neighbor slices to explore the temporal information. On each spatio-temporal attention module, the spatial information and the temporal information are connected and enhanced to recover the missing details.

The overall structure of the proposed MSBFAN model is illustrated in Fig 3. The operation of MSBFAN can be divided into three parts: Initial feature extraction, spatio-temporal attention modules and reconstruction. Firstly, the initial feature extraction module is employed to extract the shallow features of the input sequence LR images {…, *I*_*t*−1_, *I*_*t*_, *I*_*t*+1_, …} where *I*_*t*_ is the target slice. Subsequently, these shallow features are then transmitted to the spatio-temporal attention module iteratively to generate the hierarchical features {$S_{t}^{f},S_{t}^{b}$} and output SR images {*H*_*t*_}. Finally, the output SR images are collected into the reconstruction module to generate the final SR image.

#### Initial feature extraction

The initial feature extraction module consists of a 3*3 convolution layer and an activation layer. Denote *F*_*E*_(⋅) as the feature extract function, then for target slice *I*_*t*_, the shallow features *S*_*t*_ extracted can be represented as:

$$\begin{array}{c} S_{t}=F_{E}\left(I_{t}\right). \end{array}$$ (1)

For each neighbouring slice {…, *I*_*t*−1_, *I*_*t*+1_, …}, we simply concatenate the *I*_*t*_ with *I*_*t*+*k*_, the shallow features *T*_*t*+*k*_ extracted can be represented as:

$$\begin{array}{c} T_{t+k}=F_{E}(I_{t}⊕I_{t+k}),k\in \left[1,n\right]. \end{array}$$ (2)

#### Spatio-temporal attention module

**Fig 4:**
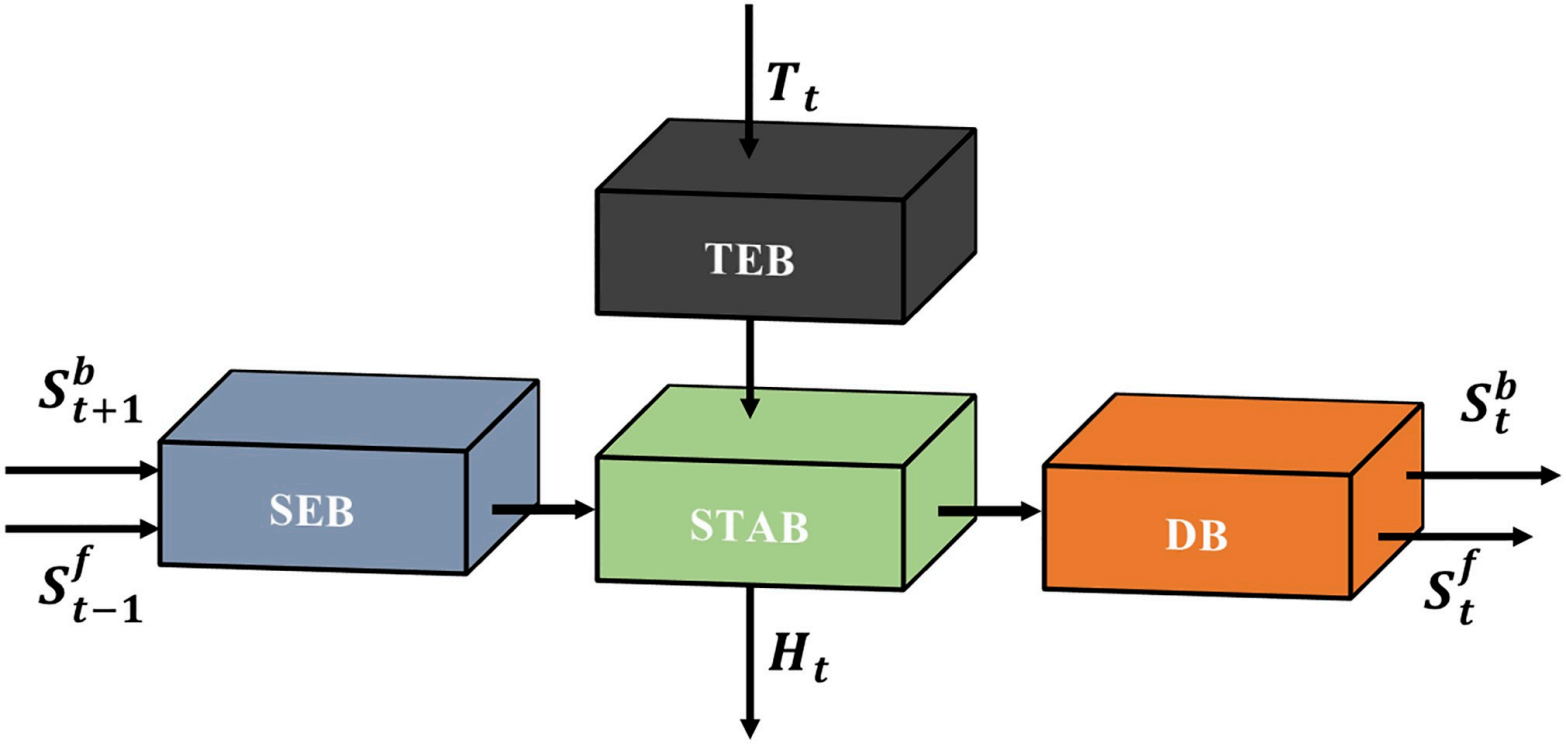
The propose spatio-temporal attention module. The spatial features of adjacent slices explored by spatial exploration block (SEB) and the temporal features of target and neighbor slices explored by temporal exploration block (TEB). Spatial and temporal features are concatenated and enhanced to construct better HR features and the next LR features produced by downsampling block (DB) for the next module.

Our proposed STAM is illustrated in Fig 4. The STAM is composed of temporal exploration block (TEB), spatial exploration block (SEB), spatio-temporal attention block (STAB) and downsampling block (DB). Here, abundant spatial information is extracted by SEB, and temporal information is extracted by TEB. We extract the missing details of the target slice by STAB, which integrates the SEB and TEB paths, then produce a refined HR feature. This part receives $S_{t-1}^{f}$, $S_{t+1}^{b}$ and *T*_*t*_, and outputs $S_{t}^{f}$, $S_{t}^{b}$ and *H*_*t*_.

*Temporal exploration block*. Similar to ResNet, we stack several residual groups which contains two residual layers to form a very lightweight network. Denote *F*_*T*_(⋅) as the TEB function, then for shallow temporal features *T*_*t*+*k*_, the output of k-th TEB can be obtained by *F*_*T*_(*T*_*t*+*k*_), *k* ∈ [1, *n*].

*Spatial exploration block*. We creatively propose a multi-scale back-projection network that stacks multi-scale projections which contain several up- and down-sampling layers to expose the different scales projection errors. Denote *F*_*S*_(⋅) as the SEB function, then for shallow spatial features *S*_*t*+*k*_, the output of k-th SEB can be obtained by *F*_*S*_(*S*_*t*+*k*_), *k* ∈ [1, *n*].

*Spatio-temporal attention block*. STAB receives and concatenates *F*_*T*_(*T*_*t*+*k*_) and *F*_*S*_(*S*_*t*+*k*_), then produces refined periodical HR features through the spatio-temporal fusion attention. Denote *F*_*A*_(⋅) as the spatio-temporal fusion attention function, the HR features *H*_*t*+*k*_ produced can be represented as:

$$\begin{array}{c} H_{t+k}=F_{A}(F_{T}(T_{t+k})⊕F_{S}(S_{t+k})),k\in \left[1,n\right]. \end{array}$$ (3)

*Downsampling block*. DB downsamples the HR features *H*_*t*+*k*_ then outputs $S_{t+k}^{f}$ and $S_{t+k}^{b}$. Denote *F*_*D*_(⋅) as the downsampling function, the $S_{t+k}^{f}$ and $S_{t+k}^{b}$ produced can be represented as:

$$\begin{array}{c} S_{t+k}^{f}=S_{t+k}^{b}=F_{D}\left(H_{t+k}\right),k\in \left[1,n\right]. \end{array}$$ (4)

Therefore, supposing the sequence medical images have *n* + 1 slices *I*_*t*+*k*_, *k* ∈ [1, *n*], and *I*_*t*_ is the target slice, then the output of the last STAM can be iteratively formulated as follow:

$$\begin{array}{c} S_{t+1}=F_{D}^{1}((F_{A}^{1}(F_{T}^{1}(T_{t+1})⊕F_{S}^{1}(S_{t}))))\;\; \end{array}$$ (5)

$$\begin{array}{c} H_{t+n}=F_{A}^{n}(\ldots (F_{A}^{2}(F_{T}^{2}(T_{t+2})⊕F_{S}^{2}(S_{t+1})))\ldots ). \end{array}$$ (6)

These periodical HR features constitute the final out of our STAMs.

#### Reconstruction

The final SR output is generated by feeding concatenated HR features for all STMs into a reconstruction module, the

$$\begin{array}{c} SR_{t}=F_{rec}\left(\left[H_{t+1}⊕H_{t+2}⊕\ldots ⊕H_{t+n}\right]\right). \end{array}$$ (7)

In our model, *F*_*rec*_ is a single convolution layer with the kernel size of 3*3.

## Experimental results

In this section, we first introduce the training dataset and implementation details. Then we compare the different configurations of MSBPN and the whole network on SR performance. Finally, our MSBFAN model is compared with several state-of-the-art SR algorithms. We evaluate the quantitative SR result with PSNR and SSIM. In all our experiments, we focus on 4x SR factor.

### Dataset and implementation details

Our training dataset is constructed of the IXI dataset which contains three subsets of MR images: 578 PD volumes, 581 T1 volumes and 578 T2 volumes. We divided the training set, testing set, and verification set in a ratio of approximately 100:10:1 for each subset. We select and clip these three types of 3D volumes to the size of 240 x 240 x 91 (height x width x depth)and then generate 47985, 51548, 51184 2D training examples and 6855, 7364, 7312 7-slices training examples, respectively. We also apply augmentation, such as flipping and rotation, to generate the LR image. We downscale the HR image with bicubic interpolation.

For TEB, we construct nine blocks where each block consists of two 3*3 convolutional layers. The up- and down-layer in TEB and DB use 8*8 kernel with stride = 4 and pad by 2 pixels. For SEB, we construct four scales (1x, 2x, 3x, 4x) projection units where each projection unit consists of three up-sampling layers and two down-sampling layers (n = 1, m = 2). For 1x projection unit, the up- and down-sampling layers use 3*3 kernel with stride = 1 and pad by 1 pixel; For 2 x projection unit, the up- and down-sampling layers use 6*6 kernel with stride = 2 and pad by 2 pixels; For 3x projection unit, the up- and down-sampling layers use 7*7 kernel with stride = 3 and pad by 2 pixels; And for 4x, the configuration of the up- and down-sampling layers same with the TEB and DB. The number of feature maps is used *c*^*t*^ = *c*^*s*^ = 64.

We train the models with patch size 48 × 48, which is cropped randomly from 60 × 60 LR images. All models are trained end-to-end using *L*_1_ loss, and the learning rate is initialized as 10^−4^ for all layers and decrease by a factor of 10 for half of the total 100 epochs. For optimization, we used Adam by setting *β*_1_ = 0.9, *β*_2_ = 0.999 and *ϵ* = 10^−8^. All experiments were conducted using Python 3.8.5 and PyTorch 1.6.0 on NVIDIA GeForce GTX 1080 Ti GPU.

### Model analysis

#### Multi-scale back-projection network

**Fig 5:**
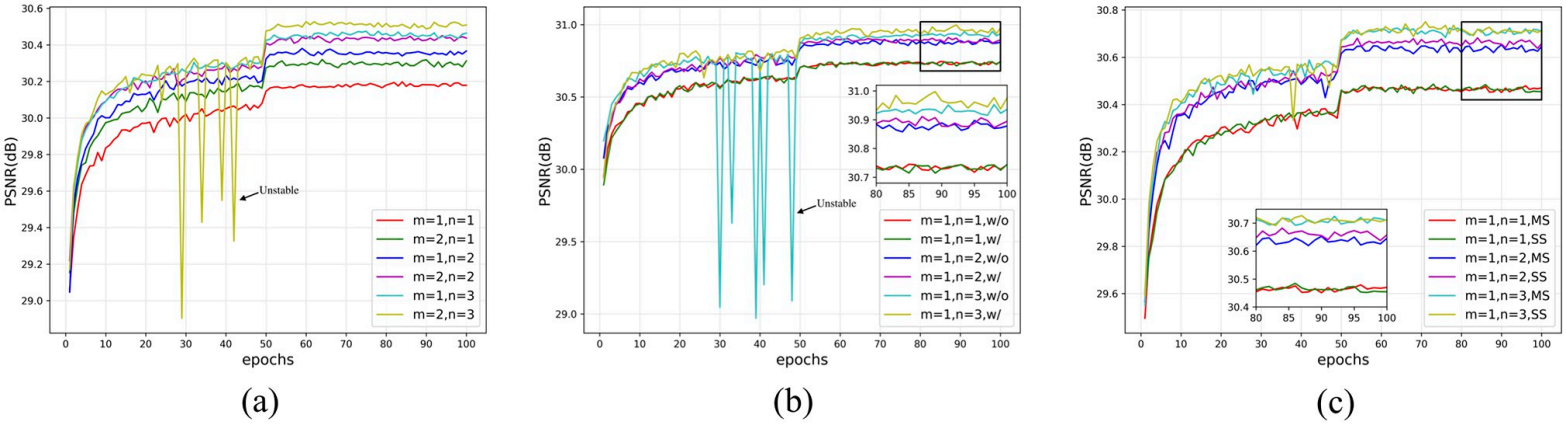
The performance comparison between different configures of our MSBPN for SR 4x. (a) Impact of projection units and the convolutional layers of the projection unit validated on PD. (b) Dense Connection, validated on T1. (c) Multi-scale machine, validated on T2.

The proposed MSBPN can be configured in several ways. For comparison, we have verified the structure of different MSBPN modules from the following aspects:

*Back-projection*. To study the impact of different configurations of back-projection, we construct multiple modules to show the tradeoff between performance and the number of network parameters. Specifically, we create two kinds of modules *M*_1,*n*_, and *M*_2,*n*_ to investigate the impact of the number of convolutional layers of the projection unit. We also created the other three kinds of modules *M*_*m*,1_, *M*_*m*,2_, and *M*_*m*,3_ to investigate the impact of the number of projection units. The training and testing results are shown in Fig 5(a) and Table 1. It can be seen that the performance of the model is improved with the deepening of the network depth which is mainly determined by the number of projection units *m* and the number of convolutional layers of projection unit *n*. We can infer that the performance improvement on our MSBPN is mainly due to the increase of model depth. However, the depth of the model does not increase indefinitely. When m = 2, n = 3, the model training began to be unstable, proving that models with complex structures and plenty of parameters are promising to improve model performance, but it is more challenging to be fully trained with MR images.

**Table 1:** The testing performance of different configurations of projection units and the convolutional layers of projection unit on PD for SR 4x.

Network Configuration	Network Parameters	Network Depth	PSNR(dB)	SSIM
*M*_*m* = 1, *n* = 1_	2,863K	16	30.82	0.9178
*M*_*m* = 1, *n* = 2_	5,480K	32	31.02	0.9211
*M*_*m* = 1, *n* = 3_	8,058K	48	31.12	0.9225
*M*_*m* = 2, *n* = 1_	4,158K	24	30.95	0.9199
*M*_*m* = 2, *n* = 2_	8,070K	48	31.10	0.9225
*M*_*m* = 2, *n* = 3_	11,943K	72	31.17	0.9234

*Dense connection*. We can remove the dense connection of the MSDPN to show how dense connection influences the performance of the model in three cases, as shown in Fig 5(b) and Table 2. Dense connection stabilizes the training deeper network and adaptively reuses the extraction of information from current and preceding back-projection units.

**Table 2:** Test results of the models with different connection approximations on T1 for SR 4x (PSNR/SSIM).

DC \ Model	*M*_*m* = 1, *n* = 1_	*M*_*m* = 1, *n* = 2_	*M*_*m* = 1, *n* = 3_
w/	29.74/0.8879	29.91/0.8917	29.98/0.8934
w/o	29.75/0.8879	29.88/0.8913	29.94/0.8926

*Multi-scale*. To demonstrate the advantage of our multi-scale mechanism, we build two kinds of networks, SS which adopt single-scale projection units (four 4x units) and MS which adopt multi-scale projection units (1x, 2x, 3x and 4x units). Those two networks were compared in terms of performance and the number of parameters. The results on 4x enlargement are shown in Fig 5(c) and Table 3. It is observed that the multi-scale machine helpful to reduce the parameters of the model significantly, and the performance is not compromised.

**Table 3:** Test results of the model with multi-scale configuration on T2 for SR 4x (PSNR/Parameters).

MS \ Model	*M*_*m* = 1, *n* = 1_	*M*_*m* = 1, *n* = 2_	*M*_*m* = 1, *n* = 3_
w/	30.12/2,863K	30.30/5,480K	30.39/8,058K
w/o	30.11/4,469K	30.31/8,691K	30.38/12,930K

#### Multi-scale bidirectional fusion attention network

In this part, we validate several components of the proposed MSBFAN and mainly focuses on temporal information usage.

*Baselines*. We consider three baselines with different spatial and temporal information fusion. First, we simplify concatenate all slices (7 slices) as the input of the SEB, which introduces temporal information but hasn’t been explored enough. Second, we remove the $S_{t}^{b}$ stream, only keep $S_{t}^{f}$, which turns off the backward temporal connection. Third, we remove the $S_{t}^{f}$ stream, only keep $S_{t}^{b}$, which turns off the forward temporal connection. The testing results are shown in the Table 4. The results of SEB (1 slice) and SEB (7 slices) show that extracted information from neighbouring slices contributes to the image reconstruction. The combination of spatial and temporal information is also important. The full MSBFAN model can achieve 31.41 dB, which is better than 0.37db, 0.08 dB and 0.09 dB than SEB(7slices), MSBFAN (forward) and MSBFAN (backward).

**Table 4:** Baseline comparison on PD for SR 4x (PSNR/SSIM).

	SEB (1 slice)	SEB (7 slices)	MSBFAN (forward)	MSBFAN (backward)	MSBFAN
PSNR	30.45	31.04	31.33	31.32	31.41
SSIM	0.9119	0.9217	0.9261	0.9260	0.9266

**Fig 6:**
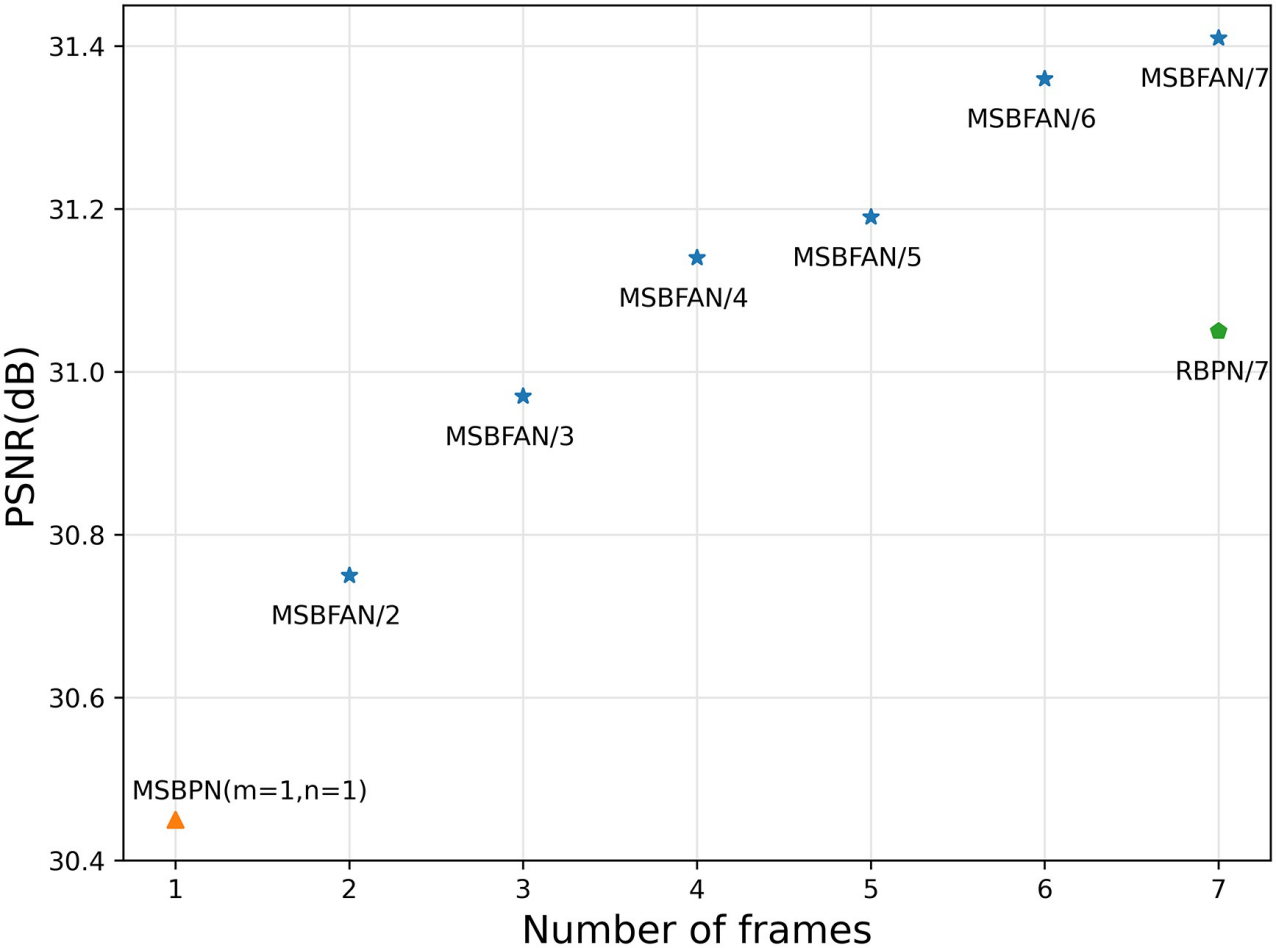
The performance comparison between the model with different number of slices, 4x SR on PD. MSBFAN/s: MSBFAN trained/tested with *s* slices. Note: MSBFAN/1 equivalent MSBPN (n = 1, m = 2).

*Slice length*. We evaluated MSBFAN with different lengths of MR image sequences. Fig 6 shows the performance improves with the more extended slices. As we can see from the figure, the model achieves the most improvement when increasing the slices from one to two and three, on account of the slices closest to the target slice have the highest correlation. The performance of MSBFAN/4 is even better than RBPN/7 which we refer to. Predictably, the performance of our MSBFAN will be further improved as the number of slices increases.

*Slice order*. When selecting the MR image sequence to serve as a slice for the target slice *I*_*t*_, we have a choice of how to choose it. We consider three case: use only past 2 slices (*I*_*t*−2_, *I*_*t*−1_), named P; use only future 2 slices (*I*_*t*+1_, *I*_*t*+2_), named F; use both past 1 slice (*I*_*t*−1_) and future 1 slice (*I*_*t*+1_), named PF. P represents the network is trained and tested on P, P → F represents the network is trained on P and tested on F. The results are shown in Table 5. Our intuition suggests, and the results confirm, that PF is better than P and F by 0.19dB, since the nearest slice has more supplementary information about the target slice. P is better than P → F by 0.25dB, and F is better than F → P by 0.21dB indicate that the MR image sequence does not have symmetry. P and F achieve similar performance indicates that the model is robust and insensitive to the order of MR image sequence.

**Table 5:** Effect of temporal order of slice on PD for SR 4x.

Test \ Train	P	F	PF
P	30.78/0.9173	30.57/0.9142	30.81/0.9184
F	30.53/0.9138	30.78/0.9171	30.63/0.9163
PF	30.73/0.9166	30.69/0.9160	30.97/0.9208

*Ablation study*. To verify the superiority of our MSBFAN, we investigate the basic network modules: the SEB and STAB. To demonstrate the effect of our SEB, we use DBPN instead of our MSBPN (denote as M→D for short). To demonstrate the effect of our STAB, we use residual learning instead our spatio-temporal fusion attention (denote as FA→RL for short). We further show the effect of optical flow (OF) on the performance of our model. Table 6 shows the ablation investigation on the effects of the three described above. When we compare the results of the second line and last line, we find that model with MSBPN would perform better than those with DBPN. Benefitting from our multi-scale machine, the performance was improved from 30.71dB to 30.82dB, while the number of parameters was reduced by nearly 16.6%. This comparison firmly demonstrates the effectiveness of MSBPN and indicates adaptive different scale image detail improves the performance. From the comparison between the third line and the last line, we can conclude that adopting our spatio-temporal fusion attention block is a better way to promote the fusion of spatio-temporal information, and the performance is improved by 0.16dB when the number of parameters is similar. When both SEB and STAB are replaced, the performance drops significantly, up to 0.25dB. Just as we predicted, the optical flow information of the slices sequence compromised the performance of the model, reducing the performance by 0.02dB while increasing the computational cost.

**Table 6:** Investigations of SEB, STAB and optical flow on T2 for SR 4x.

M → D	FA → RL	OF	PSNR/SSIM/Parameters
√			30.71/0.9184/5.683K
	√		30.66/0.9178/4.889K
√	√		30.57/0.9168/5.829K
		√	30.80/0.9190/4.742K
			30.82/0.9199/4.742K

### Comparison with other methods

To verify the effectiveness of the proposed MSBPN and MSBFAN more scientifically, we compare them with several advanced SR algorithms: VDSR [25], LapSRN [24], EDSR [21], DBPN [20], RDN [22], CSN [18], RCAN [28], SAN [45], HAN [46], Swin [47], LBNet [48], RBPN [34] and BasicVSR [49]. All models are retrained with the same training configuration on generated three datasets. Each data has a different focus and characteristics.

Table 7 shows that our MSBPN performs less than satisfactory at small scales. This is due to the small scale used less scales. For example, when the scale is 3, the scales used are x1, x2 and x3. This is done to effectively reduce the number of model parameters. Compared with RDN, our MSBPN achieves considerable performance with a small number of parameters for scale factor 4. However, all these single slice-based methods perform worse than the proposed multitude slices-based MSBFAN, indicating the proposed method’s superiority. Specifically, the PSNR value on three datasets achieved by our model is higher than HAN by 0.22dB, 0.21dB, and 0.32dB for scale factor 4, respectively. That is because, different scale spatial information can be well explored by our MSBPN and more supplementary temporal information can be aggregated into the features of the target slice, which is a great help in reconstructing high quality images. Our MSBFAN achieves better accuracy than the same multitude slices-based model RBPN and BasicVSR, even though RBPN has more than twice as many parameters. Note that RBPN and BasicVSR training failure for scale factor 2. This shows that simply applying natural image algorithms to medical images is not feasible.

**Table 7:** Quantitative comparison between the state-of-the-art SR algorithms on 3 test datasets.

Scale	Model	Parameter	Runtime	Slice	PD		T1		T2	
					PSNR	SSIM	PSNR	SSIM	PSNR	SSIM
x2	VDSR	665K	163ms	1	37.96	0.9790	36.04	0.9700	37.16	0.9746
	LapSRN	870K	171ms		37.98	0.9794	36.04	0.9703	37.13	0.9749
	EDSR	40,720K	170ms		38.55	0.9811	36.37	0.9719	37.73	0.9767
	DBPN	5,941K	175ms		38.59	0.9812	36.43	0.9722	37.73	0.9767
	RDN	22,121K	192ms		38.76	0.9817	36.52	0.9726	37.93	0.9773
	CSN	22,036K	262ms		38.95	0.9821	35.60	0.9727	38.09	0.9777
	RCAN	15,442K	242ms		39.01	0.9823	36.64	0.9730	38.13	0.9780
	SAN	15,721K	560ms		39.04	0.9824	36.67	0.9730	38.14	0.9780
	HAN	15,922K	251ms		39.06	0.9825	36.68	0.9734	38.15	0.9780
	Swin	11,748K	321ms		39.02	0.9823	36.65	0.9728	38.12	0.9780
	LBNet	-	-		-	-	-	-	-	-
	MSBPN	4,044K	167ms		38.54	0.9811	36.40	0.9720	37.70	0.9767
	RBPN	-	-	7	-	-	-	-	-	-
	BasicVSR	-	-		-	-	-	-	-	-
	MSBFAN	4,398K	278ms		39.18	0.9829	36.82	0.9740	38.36	0.9788
x3	VDSR	665K	167ms	1	32.51	0.9416	31.37	0.9217	31.97	0.9362
	LapSRN	944K	167ms		32.58	0.9428	31.41	0.9226	32.01	0.9370
	EDSR	43,671K	175ms		33.44	0.9512	31.97	0.9302	32.77	0.9445
	DBPN	8,018K	181ms		33.48	0.9513	32.01	0.9305	32.85	0.9451
	RDN	22,121K	188ms		33.56	0.9523	32.10	0.9318	32.93	0.9462
	CSN	22,037K	258ms		33.68	0.9533	32.17	0.9325	33.05	0.9472
	RCAN	15,627K	243ms		33.70	0.9534	32.19	0.9329	33.06	0.9472
	SAN	15,905K	273ms		33.71	0.9535	32.19	0.9329	33.07	0.9474
	HAN	16,106K	245ms		33.70	0.9533	32.23	0.9333	33.10	0.9477
	Swin	11,932K	318ms		33.68	0.9530	32.20	0.9330	33.10	0.9477
	LBNet	736K	143ms		33.40	0.9502	31.90	0.9292	32.70	0.9430
	MSBPN	7,183K	176ms		33.48	0.9513	32.03	0.9307	32.84	0.9449
	RBPN	11,338K	223ms	7	33.53	0.9518	32.11	0.9326	32.95	0.9459
	BasicVSR	6,325K	314ms		33.66	0.9530	32.28	0.9343	33.14	0.9478
	MSBFAN	4,557K	234ms		33.93	0.9551	32.49	0.9367	33.30	0.9487
x4	VDSR	665K	168ms	1	29.96	0.9031	29.09	0.8719	29.30	0.8955
	LapSRN	870K	164ms		30.51	0.9126	29.50	0.8823	29.85	0.9056
	EDSR	43,081K	173ms		31.02	0.9218	29.94	0.8929	30.28	0.9133
	DBPN	10,426K	176ms		31.06	0.9218	29.95	0.8927	30.34	0.9138
	RDN	22,269K	189ms		31.12	0.9230	30.03	0.8946	30.42	0.9156
	CSN	22,157K	285ms		31.14	0.9234	30.04	0.8950	30.45	0.9160
	RCAN	15,590K	244ms		31.16	0.9238	30.04	0.8951	30.44	0.9159
	SAN	15,869K	227ms		31.18	0.9240	30.06	0.8954	30.43	0.9153
	HAN	16,069K	246ms		31.19	0.9241	30.08	0.8959	30.50	0.9170
	Swin	11,896K	320ms		31.18	0.9241	30.05	0.8952	30.48	0.9166
	LBNet	742K	140ms		31.02	0.9216	29.90	0.8920	30.27	0.9130
	MSBPN	8,058K	174ms		31.12	0.9225	29.98	0.8934	30.39	0.9146
	RBPN	12,751K	215ms	7	31.05	0.9215	29.98	0.8950	30.45	0.9148
	BasicVSR	6,288K	320ms		31.24	0.9250	30.11	0.8971	30.60	0.9179
	MSBFAN	4,742K	218ms		31.41	0.9266	30.29	0.9011	30.82	0.9199

**Fig 7:**
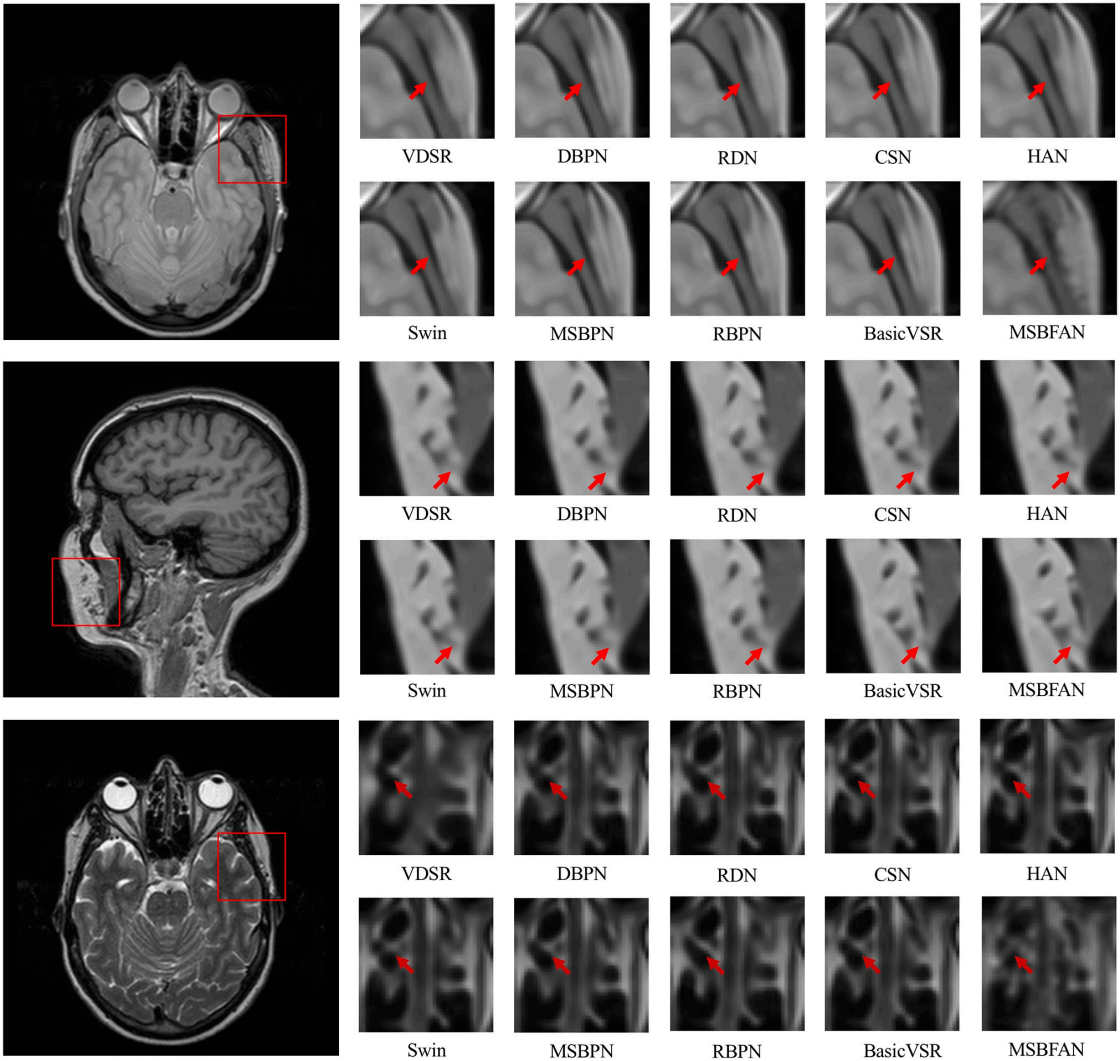
The visual effect of the compared methods on a PD (top), T1 (middle) and T2 (bottom) image with SR x4.

Fig 7 displays the qualitative results on three scenarios of the PD, T1, and T2 dataset, respectively. It can be observed from the zoom-in regions that our model reconstructs plentiful and more authentic details and it has the most similar entirety to the ground truth. The first and second rows show the result of a PD image. There is a lot of texture at the position indicated by the red arrow, which not completely be reconstructed in the results of other models, but our model gives a relatively comprehensive and clear reconstruction result. The third and fourth rows show the result on a T1 image. Similar to PD, there is a black ridge at the position indicated by the red arrow, which divide the area into smaller areas. Only our MSBFAN can restore this area well. The last two lines show the result of a T2 image. It can be observed that our MSBFAN has almost successfully restored the black and white area. However, several other methods, such as CSN, HAN, Swin and BasicVSR lost the corresponding area.

## Conclusion

In this work, we have proposed a novel multi-scale back-projection network (MSBPN) for a single target MR image, primarily made up of different scale back-projection units to extract abundant spatial information. Inspired by video super-resolution, we also presented a multi-scale bidirectional fusion attention network (MSBFAN) to integrating the spatial information and temporal information of sequential medical images. The temporal information is explored from the medical image sequence surrounding the target slice and iteratively integrated with the spatial information, yielding gradual refinement of the high-resolution features used, eventually, to reconstruct the high-resolution target slice. In extensive experiments, we verify the various design in the ultimate performance of our model and demonstrate that, on the IXI dataset, MSBFAN achievements significantly performance advantages over most existing SR methods.
